# Integrating clinical indications and patient demographics for multilabel abnormality classification and automated report generation in 3D chest CT scans

**DOI:** 10.3389/fradi.2025.1672364

**Published:** 2025-10-24

**Authors:** Theo Di Piazza, Carole Lazarus, Olivier Nempont, Loic Boussel

**Affiliations:** ^1^ UCBL, INSA Lyon, CNRS, INSERM, CREATIS UMR 5220, U1294, Villeurbanne, France; ^2^ Department of Radiology, Croux-Rousse Hospital, Hospices Civils de Lyon, Lyon, France; ^3^ Philips Clinical Informatics, Innovation Paris, Paris, France

**Keywords:** abnormality classification, report generation, multimodal, 3D CT scans, clinical indications, patient demographics

## Abstract

The increasing number of computed tomography (CT) scan examinations and the time-intensive nature of manual analysis necessitate efficient automated methods to assist radiologists in managing their increasing workload. While deep learning approaches primarily classify abnormalities from three-dimensional (3D) CT images, radiologists also incorporate clinical indications and patient demographics, such as age and sex, for diagnosis. This study aims to enhance multilabel abnormality classification and automated report generation by integrating imaging and non-imaging data. We propose a multimodal deep learning model that combines 3D chest CT scans, clinical information reports, patient age, and sex to improve diagnostic accuracy. Our method extracts visual features from 3D volumes using a visual encoder, textual features from clinical indications via a pretrained language model, and demographic features through a lightweight feedforward neural network. These extracted features are projected into a shared representation space, concatenated, and processed by a projection head to predict abnormalities. For the multilabel classification task, incorporating clinical indications and patient demographics into an existing visual encoder, called CT-Net, improves the F1 score to 51.58, representing a +Δ6.13% increase over CT-Net alone. For the automated report generation task, we extend two existing methods, CT2Rep and CT-AGRG, by integrating clinical indications and demographic data. This integration enhances Clinical Efficacy metrics, yielding an F1 score improvement of +Δ14.78% for the CT2Rep extension and +Δ6.69% for the CT-AGRG extension. Our findings suggest that incorporating patient demographics and clinical information into deep learning frameworks can significantly improve automated CT scan analysis. This approach has the potential to enhance radiological workflows and facilitate more comprehensive and accurate abnormality detection in clinical practice.

## 1 Introduction

Three-dimensional computed tomography (3D CT) scans have become essential tools in medical imaging [1], offering unparalleled insights into anatomical structures and pathological conditions. This type of medical image is critical for identifying diseases such as pleural effusion [2], lung cancer [3], and cardiomegaly [4]. Given the rapidly growing number of scans to analyze [5] and the increasing demand for specialized radiological expertise in many healthcare systems [6, 7], automating abnormality classification has emerged as an active research area [8–10] to enhance radiologist efficiency. The interpretation of 3D CT scans presents a time-intensive challenge, exacerbated by the heterogeneous nature of observed anomalies. Some anomalies, such as lung nodules [11], can be very small, requiring careful attention from radiologists to avoid missing them. Hence, depending on the patient demographics [12] and clinical indications [13], radiologists may dedicate more time to specific anatomical regions that could potentially present anomalies. As illustrated in Table 1, clinical indications consists of a brief paragraph written by the radiologist before the examination, describing the patient’s condition, reason for the visit, and any suspected pathologies that might be revealed during the examination.

**Table 1:** Examples of patient demographics (sex and age) and clinical indications from the CT-RATE dataset [10].

ID	Sex	Age	Clinical indications
Patient 1	F	64	Shortness of breath
Patient 2	F	42	Suspicion of lung cancer
Patient 3	M	50	Hematological malignancy fever chest pain
Patient 4	M	37	Patient with multiple myeloma, focus of infection

**Figure 1:**
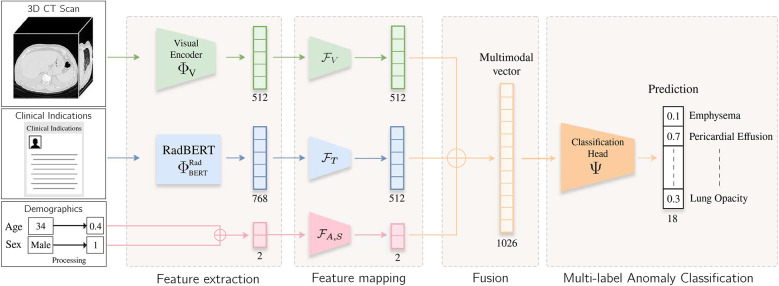
Overview of the method. The input volume is processed by a visual extractor $\Phi_{V}$ [either CT-Net [9] or CT-ViT [19]] and $F_{V}$, which generates a visual embedding. Clinical indication is processed by RadBERT [20], yielding a token-level embedding. The [CLS] token is fed into a lightweight MLP $F_{T}$ to project textual and visual features into a common latent space. Patient age and sex information are processed by another lightweight MLP $F_{A,S}$. These vectors are concatenated, and the resulting vector is passed to a classification head Ψ, which predicts an abnormality score for each label.

Inspired by the workflow of radiologists, we propose a multimodal end-to-end model that integrate clinical indications, patient age, and sex to predict chest pathologies [14]. As shown in Figure 1, our approach extends state-of-the-art methods relying on 3D CT scans to the integration of textual data corresponding to clinical indications, along with utilizing structured data such as patient age and sex. These data have a significant impact on the prevalence of a pathology [15, 16]. We separate feature extraction from each modality using individual modules and then aggregate all these extracted features to predict anomalies. As illustrated in Figure 3, we extend our experimental results by leveraging this multimodal encoder to enhance existing automated report generation methods [17, 18]. Our contributions are as follows:

• We introduce a supervised multimodal method for multilabel classification, capable of taking the 3D CT scan, clinical indications, age, and sex as input.
• We evaluate the model on a public dataset and add an ablation study to demonstrate the importance of each module.
• We extend our experimental results by integrating clinical indications and patient demographics into the automated report generation task.

## 2 Related work

### 2.1 Supervised abnormality classification

In the domain of abnormality classification in medical imaging [21], significant research has been conducted on 2D imaging [22, 23] across various modalities such as magnetic resonance imaging (MRI) [24, 25], x-rays [26–29], and skin images [30]. In the field of x-ray imaging, the publicly available MIMIC-CXR dataset [31], comprising 2D radiographs and associated clinical reports, has facilitated the development of various supervised approaches for abnormality detection [32–35] and classification [36]. While some methods focus on a single abnormality or a specific anatomical region [37, 38], others adopt a more comprehensive approach by aiming to simultaneously detect or classify multiple anomalies [39–41] using deep learning models. However, new challenges emerged with 3D imaging and the use of CT or 3D MRI. These modalities introduce novel challenges stemming from the scarcity of publicly available datasets in this domain [10], the high-dimensional nature of the data, and the significant computational demands. Prior work [9, 42] adopted traditional convolutional neural network (CNN) architectures. Recent advances have adopted transformer-based architectures [43] for volumetric data analysis. ViViT [44], an extension of the Vision Transformer [45] originally designed for video understanding [46], has demonstrated strong representational capacity and has since been adapted for a range of CT-based tasks, including radiology report generation [18] and synthetic volume generation [19], with the introduction of CT-ViT [19], which has already demonstrated its effectiveness for various tasks such as report generation [18] and abnormality classification [10].

### 2.2 Multimodal fusion

In machine learning, multimodal fusion [47] has played a pivotal role in advancing classification tasks across various research domains [48–51]. By integrating information from multiple data sources or modalities [52–54], such as combining images from different imaging techniques (e.g., MRI, CT, PET) or fusing imaging data with clinical records [55] or biological information [56], multimodal approaches offer significant advantages. They not only enhance the discriminative capability of classification models but also provide resilience against the inherent variability in single-modal datasets [47, 57, 58]. Feature extraction from each modality is typically performed using a module per modality [59] and then aggregated with a fusion module [60, 61]. The fusion of features across modalities can be achieved through simple concatenation [48], by leveraging self-attention mechanisms [59], or via cross-modality attention modules[62]. Regarding specific work on 3D CT scans, CT2RepLong [18] automatically generates a medical report from the volume and imaging report of the previous medical report of the same patient. This fusion between visual and textual features is achieved through a cross-attention module.

### 2.3 Report generation

Image captioning [63] refers to generating textual descriptions from input images, with significant progress made across various application domains [64–66]. In medical imaging, early generation methods [67] were introduced for 2D modalities using public datasets, such as x-rays [31]. The initial approaches, based on encoder–decoder architectures [68], extract a vector representation using a visual encoder (typically a CNN or attention-based model) and then pass it to a decoder module, often relying on attention mechanisms, to generate the report. Recently, the incorporation of relational memory [69], prior knowledge [70], large language models (LLMs) [71], reinforcement learning [72], and guidance-based methods [73] has enhanced the quality of generated reports. Existing methods for x-ray report generation [74] incorporate medical knowledge or prior information, often in the form of textual modalities, to enhance the quality of the generated reports [75–77]. For 3D CT volumes, the CT-RATE public dataset [10] enabled the development of CT2Rep [18], the first end-to-end method for report generation that extracts vector representations from CT-ViT [19] and passes them to a decoder to generate the report. Similar to 2D imaging, integrating LLMs [78] or multiview encoders [79] has shown improvements in report quality. In the 2D x-ray imaging domain, prior works have explored the integration of clinical indications for report generation. For example, SEI and MLRG employ cross-attention mechanisms to combine indication features with multiview or historical case information [80, 81], while Pragmatic LLaMA introduces indications as additional input to a large language model for guiding report generation [82]. These approaches share with our work the idea of leveraging clinical indications to enrich textual output. However, they are designed for 2D chest radiographs, whereas our method targets volumetric 3D CT scans, which present unique challenges in terms of data dimensionality, abnormality diversity, and multimodal fusion. Regarding guided methods for 3D CT scans, CT-AGRG [17] decomposes the task into two steps: first, a visual encoder performs feature extraction and abnormality classification, and second, a GPT-2 model [83] fine-tuned on a medical corpus [84] generates a description for each detected abnormality. In our work, we extend these approaches by integrating clinical indications and patient demographics into CT2Rep (an end-to-end method) and CT-AGRG (a guided method) to improve performance on the report generation task.

## 3 Dataset

**Figure 2:**
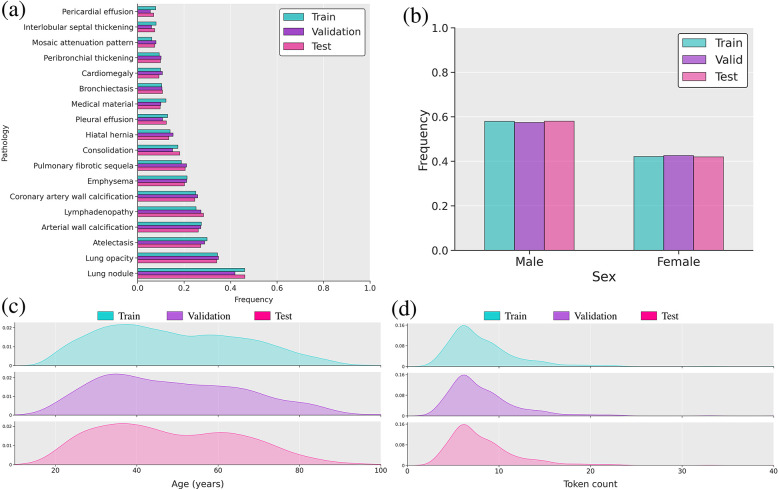
Overview of the multimodal dataset. (**a**) Bar plot of label frequency. (**b**) Bar plot of sex frequency. (**c**) Distribution of age in years. (**d**) Distribution plot of reports’ lengths based on token count using the RadBERT tokenizer.

We used the CT-RATE public dataset [10], containing 50,188 reconstructed non-contrast 3D chest CT volumes, to train and evaluate our method. For each scan, we had access to age, sex, and 18 distinct types of abnormalities. The pseudo-labels were extracted from radiology reports using a RadBERT classifier [10, 20]. We only retained samples containing clinical indications, resulting in a dataset comprising 16,009 unique patients (24,085 volumes) for the train set, 792 patients (1,551 volumes) for the validation set, and 792 patients (1,531 volumes) for the test set. We ensured there was no overlap of patients between the training, validation, and test sets. Following Draelos et al. and Hamamci et al. (author?) [9, 10], all volumes were either center-cropped or padded to achieve a resolution of 240×480×480 with in-slice spacings of 0.75 mm and 1.5 mm on the z-axis. Hounsfield unit (HU) [85] values were clipped between −1,000 and +200. Subsequently, we normalized the clipped HU values to the range [−1,1] to facilitate network training. The input age was min–max-normalized [86] to the range [0, 1] to ensure proper handling by the neural network. Sex was encoded as a binary variable, with 0 representing female and 1 representing male. Figure 2 illustrates the distribution of patient age and sex, along with the 18 abnormalities.

## 4 Methods

As illustrated in Figure 1, our feature extraction module consists of three key components. First, low-level feature extraction is performed independently for each modality, producing modality-specific vector representations. These embeddings are then mapped into a shared feature space using lightweight feedforward networks. Finally, the transformed representations are aggregated via summation to obtain a unified vector representation.

### 4.1 Visual feature extraction

The model receives an input volume $x\in R^{240\times 480\times 480}$. This volume is passed to a visual extractor $\Phi_{\text{V}}$, which is either CT-Net [9] or ViViT [19]. To demonstrate the flexibility and generality of our framework across different visual encoders, we conducted experiments using both CT-Net and ViViT. CT-Net consists of 2D ResNet [87] modules followed by a lightweight 3D convolutional network that aggregates the features maps into a compact vector representation [88]. ViViT [44] is a Vision Transformer [45] based on the attention mechanism [43] computed from 3D patches extracted from the initial volume. To ensure a fair evaluation across methods, ViViT is initialized via weight inflation [89] from a 2D ViT [45] pretrained on ImageNet [90], while the 2D ResNet module in CT-Net is directly initialized from a 2D ResNet pretrained on ImageNet. While our contribution focuses on integrating modalities such as clinical indications and demographic information into a visual encoder, we leveraged pretrained weights to facilitate network training, ensuring that model parameters are initialized under comparable conditions. Exploring alternative initialization or pretraining strategies is left for future work. From the initial volume x, both CT-Net and CT-ViT yield a vector representation $h\in R^{512}$. Subsequently, this embedding is passed to a projection head [91] $F_{V}$ to obtain $e_{V}\in R^{512}$, as defined in Equation 1 such that:

(1) $$e_{V}=F_{V}(h)=(F_{V}\circ \Phi_{\text{V}})(x)\;.$$

### 4.2 Clinical indication feature extraction

To extract embedded tokens from the textual clinical indication report, a pretrained RadBERT [20] model is used. It is a bi-directional neural network, trained on a large radiology report database on a masked language modeling task. From T tokens of the clinical indications report, a single vector representation $e_{\text{[ CLS] }}^{768}\in R^{768}$ is extracted from the Classification [CLS] token [92, 93] outputted by the language model. Working exclusively with the [CLS]-embedded token enables easy projection of textual and visual embeddings into the same-dimensional latent space. Next, $e_{\text{[ CLS] }}^{768}$ is passed through a lightweight multilayer perceptron (MLP) $F_{T}$ to project the vector representation from textual latent space of dimension 768 to a latent space of dimension 512. The resulting vector $e_{\text{[ CLS] }}\in R^{512}$ is obtained as defined by Equation 2:

(2) $$e_{\text{[ CLS] }}=F_{T}(e_{\text{[ CLS] }}^{768}).$$

### 4.3 Age and sex feature extraction

To handle the normalized age feature $x_{A}\in [0,1]$ and the sex feature $x_{S}\in \{0,1\}$, a lightweight MLP $F_{A,S}$, implemented as a linear projection followed by a ReLU activation function, is used to obtain a vector representation $e_{A,S}\in R^{2}$, as defined by Equation 3:

(3) $$e_{A,S}=F_{A,S}(x_{A},x_{S}).$$

### 4.4 Multimodal fusion

The three vector representations associated with different modalities are concatenated [54, 94] into a single vector $e\in R^{1026}$, such that $e=[e_{V},\;e_{\left[CLS\right]},\;e_{A,S}]$. A normalization layer [95] is incorporated to ensure stability during training and that the resulting vector e is properly scaled and balanced across its dimensions.

#### 4.4.1 Multilabel classification

In the context of abnormality prediction from CT scans, leveraging clinical indications and patient demographics, vector e is given a traditional classification head Ψ to obtain $\hat{y}\in R^{18}$. As commonly practiced, the model is trained on a multilabel classification task using a binary cross-entropy loss function [96].

#### 4.4.2 Report generation

**Figure 3:**
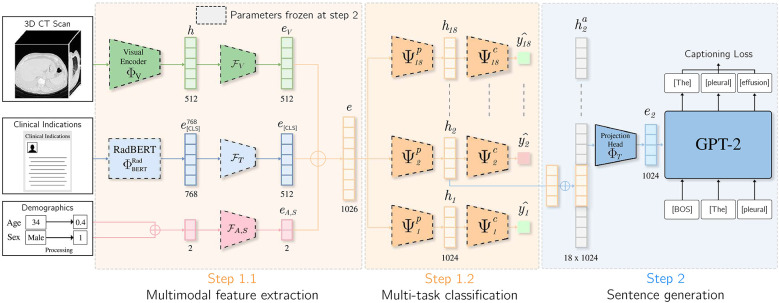
Integration of clinical indications and patients demographics for the CT-AGRG method. Features derived from the 3D CT volume, clinical indications, patient age, and sex are aggregated to form vector e. This vector is fed into 18 classification heads (one per abnormality). If a classification head predicts an abnormality, the corresponding vector representation is passed to a pretrained GPT-2 model, which generates a textual description of the detected abnormality.

To integrate clinical indications and patient demographics into the report generation task, we extended the CT2Rep [18] and CT-AGRG [17] models by replacing their original visual encoder with our proposed module, which fuses multiple modalities. As illustrated in Figure 3, the decoder responsible for generating the report takes the vector representation e as input. In CT2Rep, the decoder generates the entire report in a single pass from e. In contrast, CT-AGRG follows a two-step process: the encoder first predicts the set of abnormalities, and the decoder then generates a detailed description for each predicted abnormality. The models are trained using a next-token prediction objective with binary cross-entropy loss [96]. During inference, the decoder receives only the vector representation e of the input volume and a Beginning Of Sentence [BOS] token to signal the start of the sequence [92]. The report is then generated iteratively, token by token [83].

## 5 Experimental setup

### 5.1 Training details

For the multilabel classification task, the model was trained on 40 epochs on a GPU with 48GB of memory. We used Adam Optimizer [97] with a learning rate of $10^{-4}$ and a batch size of 4. For the report generation experiments, we adopted the same setup as used for CT2Rep [18] and CT-AGRG [17].

### 5.2 Language model

We limited the maximum number of tokens to 40, which is typically found in clinical indication reports [98]. During training, we only fine-tuned the last three layers of RadBERT, with the rest frozen [99].

## 6 Experimental results

This section is organized as follows: we first present quantitative results on the multilabel abnormality classification task with the integration of clinical indications and patient demographics; we then conduct an ablation study to assess the contribution of each module; and finally, we extend our analysis to automatic report generation.

### 6.1 Multilabel classification task

We evaluated the model’s performance using commonly used metrics: AUROC, F1 score, precision, recall, and accuracy. We also reported the weighted F1 score, computed by averaging the F1 score of each abnormality, weighted by its occurrence frequency. Because the dataset is dominated by normal findings for most labels (Figure 2), we determined label-specific thresholds on the validation set by maximizing the F1 score [100], as it balances precision and recall [21, 101]. On the test set, we then computed the average of each metric across all labels.

Table 2 demonstrates that incorporating clinical indications and patient demographics significantly improves upon state-of-the-art single-modality methods. Specifically, our model achieves an AUROC of 81.51 (+Δ3.23% over CT-Net) and the highest accuracy of 79.48. However, in an imbalanced multilabel setting, accuracy is primarily driven by correct predictions on abundant classes (especially the normal class) and therefore tends to overestimate overall performance. This also explains why precision (43.93) and recall (65.37) are lower despite high accuracy: even a small number of false positives can markedly reduce precision for rare classes. For this reason, we emphasize the F1 score as a more informative indicator of abnormality detection. Specifically, we achieved an average F1 score of 51.58 [improvements of +Δ6.13% and +Δ16.22% over CT-Net [9] and CT-ViT [10], respectively]. CT-Net with demographics and clinical indications outperforms baseline CT-Net and CT-ViT (paired t-test, p<0.01) for all metrics, indicating that incorporating clinical and demographic information enhances classification performance.

**Table 2:** Quantitative evaluation of the multilabel classification task on the test set.

Method	AUROC	Accuracy	F1 score	W. F1 score	Precision	Recall
Random predictions	49.93 ± 0.51	50.11 ± 0.37	27.18 ± 0.35	33.02 ± 0.39	20.24 ± 0.28	49.68 ± 0.51
**CT-ViT** [10]	75.14 ± 0.51	73.52 ± 0.57	44.38 ± 0.18	49.56 ± 0.25	35.35 ± 0.51	62.42 ± 0.96
**+ clinical ind. + demographics**	76.09 ± 0.37	74.83 ± 0.81	45.51 ± 0.24	50.97 ± 0.40	36.75 ± 0.43	63.00 ± 0.98
**CT-Net** [9]	*78.96* ± 0.30	*78.49* ± 0.55	*48.60* ± 0.37	*54.18* ± 0.55	*42.56* ± 1.01	*60.15* ± 0.99
**+ clinical ind. + demographics**	**81.51** ± 0.26	**79.48** ± 0.42	**51.58** ± 0.54	**57.60** ± 1.06	**43.93** ± 0.77	**65.37** ± 0.88

Reported mean and standard deviation metrics were computed over a fivefold cross-validation. The weighted F1 score corresponds to the average of F1 scores for each abnormality, weighted by the frequency of occurrence of the abnormality in the test set. **Best** results are in bold, and *second best* are in italics.

**Figure 4:**
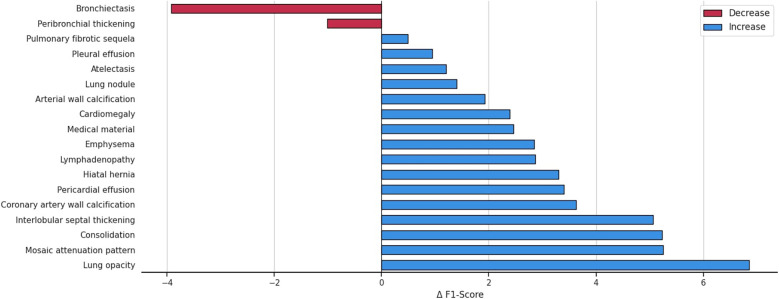
Variation in the F1 score across anomalies, highlighting the impact of integrating patient demographics and clinical indications into the multilabel abnormality classification task.

Figure 4 details the impact on F1 score for each abnormality when integrating patient demographics and clinical indications, demonstrating that this additional contextual information improves performance for 16 out of 18 anomalies. The largest gains, observed for interlobular septal thickening, consolidation, mosaic attenuation, and lung opacity, suggest that these findings are particularly context-dependent and strongly correlated with clinical factors. While most anomalies benefit from the auxiliary information, a minority, such as bronchiectasis, shows slight performance decreases, possibly because the added inputs may introduce noise for anomalies that already possess distinctive visual signatures. A promising future direction is to develop adaptive integration strategies that selectively incorporate contextual information when it is beneficial.

### 6.2 Ablation study

We conducted a comprehensive ablation study to assess the contributions of the clinical indication feature extractor, each auxiliary input modality, and the fusion module to overall performance.

#### 6.2.1 Impact of the clinical indication encoding module

**Figure 5:**
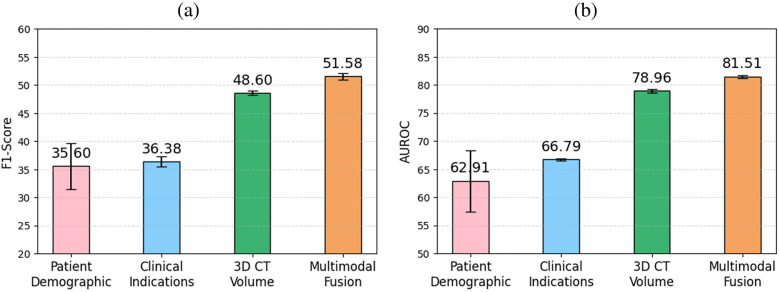
Comparison of (**a**) F1 score and (**b**) AUROC across clinical indications, patient demographics, 3D CT volume, and multimodal fusion for multilabel abnormality classification. The highest performance is achieved when fusing all modalities, highlighting the benefit of multimodal integration.

To evaluate the impact of different modules for encoding clinical indications into vector representations, we conducted an ablation study comparing three approaches: a transformer encoder trained from scratch, a BERT language model pretrained on a general corpus, and RadBERT, a BERT-based model pretrained specifically on radiology text. Table 3 and Figure 5 report the classification performance achieved when using only clinical indications as input for each of these modules. RadBERT achieved an F1 score of 36.38, representing a +Δ3.47% improvement over general-domain BERT and a +Δ4.33% improvement over the transformer encoder trained from scratch. These results suggest that leveraging a domain-specific pretrained language model facilitates the extraction of more meaningful features from clinical indications, ultimately enhancing classification performance.

**Table 3:** Comparative analysis of individual modalities and full integration for multilabel abnormality classification from 3D CT volumes.

Method	AUROC	Accuracy	F1 score	W. F1 score	Precision
Random predictions	49.93 ± 0.51	50.11 ± 0.37	27.18 ± 0.35	33.02 ± 0.39	20.24 ± 0.28
Patient demographics					
Age + sex	62.92 ± 5.46	50.83 ± 19.92	35.60 ± 4.13	43.26 ± 3.69	25.71 ± 5.24
Clinical indications					
Transformer encoder [43]	65.64 ± 0.40	64.30 ± 1.13	34.87 ± 0.24	41.80 ± 0.26	26.49 ± 0.63
BERT [92]	65.96 ± 0.07	63.28 ± 0.48	35.16 ± 0.29	42.00 ± 0.18	25.98 ± 0.41
RadBERT [20]	66.79 ± 0.21	65.41 ± 1.07	36.38 ± 0.91	42.34 ± 0.14	27.64 ± 0.64
3D CT volumes					
CT-Net [9]	*78.96* ± 0.30	*78.49* ± 0.55	*48.60* ± 0.37	*54.18* ± 0.55	*42.56* ± 1.01
Multimodal fusion					
CT-Net + RadBERT + age + sex	**81.51** ± 0.26	**79.48** ± 0.42	**51.58** ± 0.54	**57.60** ± 1.06	**43.93** ± 0.77

Results are shown for (1) patient demographics only, (2) clinical indications only, (3) visual 3D volumes only, and (4) integration of all inputs.

Bold values show “best results”.

#### 6.2.2 Impact of auxiliary information

Table 4 presents the ablation study evaluating the incremental impact of incorporating patient demographics and clinical indications as auxiliary inputs alongside the 3D CT Volumes. Adding patient demographics yields an F1 score of 49.79, reflecting a +Δ2.45% improvement over the CT-Net baseline. Incorporating clinical indications results in an F1 score of 50.86, corresponding to a +Δ4.45% gain. For each auxiliary input configuration, a paired t-test comparing the F1 score distributions against the baseline yields a p-value <0.01, highlighting the statistical significance of the observed performance improvements. Removing CT features led to a consistent drop in performance, indicating that the model does not rely solely on clinical text or metadata.

**Table 4:** Ablation study on the contribution of auxiliary information for multilabel abnormality classification from 3D CT volumes.

3D CT volumes	Patient demographics	Clinical indications	AUROC	F1 score	Paired t-test p-value	Training time	Inference time
✓			78.96 ± 0.30	48.60 ± 0.37	–	15.09 ± 1.55	4.16 ± 0.68
✓	✓		80.51 ± 0.49	49.79 ± 0.69	<0.01	16.05 ± 1.06	4.11 ± 0.32
✓		✓	*81.00* ± 0.42	*50.86* ± 0.36	<0.01	111.30 ± 3.33	76.19 ± 0.79
✓	✓	✓	81.51± 0.26	51.58 ± 0.54	<0.01	112.96 ± 4.38	76.76 ± 0.97

We report performance using (1) visual encoder alone, (2) integration of patient demographics, (3) integration of clinical indications, and (4) integration of both. The paired t-test p-value column reports the statistical significance of the F1 score improvements compared to the baseline using only 3D CT volumes. Training time and inference time indicate the average time per sample (in ms) for forward and backward passes (training) and for inference, respectively.

Bold values show “best results”.

#### 6.2.3 Impact of the fusion module

Our ablation study results related to the fusion module, presented in Table 5, indicate that concatenating features yields the highest AUROC and F1 score increase seen through the integration of clinical indications. Specifically, we obtained an F1 score of 50.86, demonstrating a +Δ0.97 improvement over sum and a +Δ2.12% improvement over cross-modality attention. This suggests that, in our specific setting, direct concatenation provides a strong signal without the overhead of more complex interaction modeling where the modalities may have relatively low complexity. While more expressive mechanisms such as cross-attention mechanisms demonstrate robust performances in large-scale multimodal learning, we found that in our setting, where the dataset is relatively modest, a simpler fusion provides more robust performance, requiring fewer parameters to fully benefit from modalities effectively. We evaluated an alternative fusion strategy where clinical indications and demographic features are combined into a prompt for the BioMistral LLM [102]. As presented in Table 6, independent concatenation of modality-specific embeddings demonstrates a +Δ0.89% improvement in the F1 score and a +Δ1.05% improvement in AUROC over the LLM-based prompt fusion. We attribute this improvement to the robustness of simpler fusion in the context of our relatively small dataset. While prompt-based fusion offers more expressive modeling, it may require larger datasets to fully realize its benefits, highlighting the importance of matching fusion complexity to dataset scale.

**Table 5:** Impact of the aggregation module between features extracted by a visual encoder from the 3D CT volumes and those extracted by RadBERT from clinical indications.

Method	AUROC	Accuracy	F1 score	W. F1 score	Precision
Random predictions	49.93 ± 0.51	50.11 ± 0.37	27.18 ± 0.35	33.02 ± 0.39	20.24 ± 0.28
CT-Net [9]	78.96 ± 0.30	78.49 ± 0.55	48.60 ± 0.37	54.18 ± 0.55	42.56 ± 1.01
Methods below utilize clinical indications					
With self-attention	80.35 ± 0.18	78.67 ± 0.78	49.21 ± 0.57	55.35 ± 0.48	42.23 ± 0.59
With cross-attention	80.36 ± 0.41	78.11 ± 1.02	49.84 ± 0.31	55.44 ± 0.27	42.29 ± 0.91
With sum	*80.99* ± 0.28	*78.89* ± 0.65	*50.37* ± 0.50	*56.08* ± 0.36	*43.05* ± 0.25
With concatenation	**81.00** ± 0.42	**79.80** ± 0.37	**50.86** ± 0.36	**56.58** ± 0.20	**43.84** ± 0.32

Bold values show “best results”.

**Table 6:** Comparison of fusion strategies for incorporating clinical indications and demographic information in 3D chest CT abnormality classification.

Demographics and indications	Fusion strategy	AUROC	Accuracy	F1 score	W. F1 score	Recall
✗		78.96 ± 0.30	78.49 ± 0.55	48.60 ± 0.37	54.18 ± 0.55	60.15 ± 1.39
✓	Prompt-based	80.66 ± 0.45	*79.68* ± 0.47	51.12 ± 0.51	56.78 ± 0.37	63.50 ± 1.28
✓	Modality-specific	*81.51* ± 0.26	79.48 ± 0.42	*51.58* ± 0.54	*57.60* ± 1.06	*65.37* ± 1.53

We evaluated modality-specific embeddings for clinical indications, patient age, and sex, concatenated with CT features, against prompt-based fusion, where the same information is integrated into a BioMistral-7B input prompt. *Best results* are underlined.

### 6.3 Report generation task

We extend our experiments to the task of automated report generation by integrating clinical information for two methods: CT2Rep [18], which generates the entire report in a single pass, and CT-AGRG [17], which first predicts abnormalities and then generates a description for each detected abnormality. Once the report is generated, we evaluate its quality using two sets of metrics: natural language generation (NLG) metrics and clinical efficacy (CE) metrics [69, 73]. NLG metrics assess the similarity between the generated text and the ground truth. We used BLEU-1 [103], which compares the overlapping 1-grams between the reference and the prediction. ROUGE evaluates recall-oriented metrics, like overlap and precision, between n-grams. BERTScore [104], an embedding-based metric, measures the cosine similarity of vector representations of the embedded tokens between the reference and the generated text. Clinical efficacy metrics evaluate the clinical accuracy of generated reports. We extracted abnormality mentions as one-hot vectors using a RadBERT language model classifier [20], which was originally used for CT-RATE [10] label annotation. These predictions are then compared to ground-truth labels using standard multilabel classification metrics, such as the F1 score. In addition, we report the CRG score [105], a recently proposed distribution-aware metric for radiology report generation. Unlike conventional metrics, CRG focuses exclusively on clinically relevant abnormalities explicitly described in the reference report, while also accounting for class imbalance.

**Figure 6:**
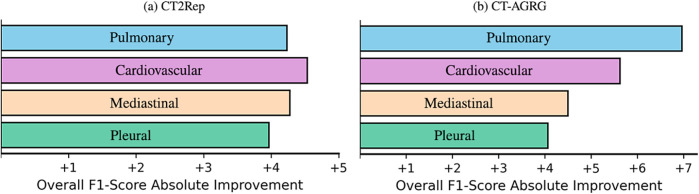
F1 score improvements across four abnormality groups, when incorporating demographic and clinical indication information into 3D CT volume report generation.

**Figure 7:**
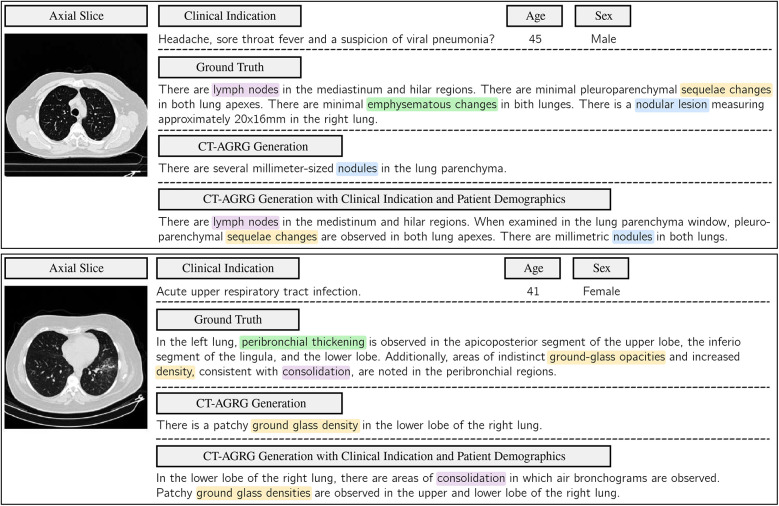
Comparison of ground-truth labels with the report generated by the CT-AGRG model with and without the integration of clinical indications and patient demographics. For each of the two CT-RATE test set examples, we present an axial slice, clinical indications, demographic information, ground truth, and the generated report. Clinical relevance is highlighted using color-coded annotations.

As shown in Table 7, the integration of clinical indications and patient demographics significantly enhances both NLG and CE metrics. For the CT2Rep model, incorporating these additional data results in a BLEU-1 score of 0.342, reflecting a +Δ7.55% increase compared to the baseline model, and an F1 score of 36.57, which corresponds to a +Δ14.78% improvement over the baseline. Similarly, for the CT-AGRG guided method, the inclusion of clinical indication and patient demographic information leads to a performance boost, achieving a recall of 60.43 (+Δ12.32% increase) and an F1 score of 48.30 (+Δ6.69% increase) over the original model. For each method, we performed a paired t-test comparing the F1 score obtained with and without the integration of clinical indications and patient demographics. The resulting p-values are all strictly below 0.01, indicating statistically significant improvements in the quality of the generated reports. In addition, Figure 6 allows us to identify which anomalies benefit most from richer multimodal inputs, making performance gains more clinically interpretable and highlighting where report generation is most reliable. Figure 7 illustrates two examples of report generation compared to the ground truth, emphasizing that our method produces reports with a structure and terminology closely resembling those written by radiologists.

**Table 7:** Quantitative evaluation of the report generation task.

Method	NLG metrics			CE metrics		
	BLEU-1	ROUGE_L_	BERT	CRG	Recall	F1 score
Random predictions	–	–	–	0.397 ± 0.004	50.92 ± 1.48	27.18 ± 0.35
CT2Rep [18]	0.318 ± 0.007	0.236 ± 0.006	0.863 ± 0.002	0.417 ± 0.009	32.56 ± 2.62	31.86 ± 1.74
**+ clinical indications + demographics**	0.342 ± 0.006	0.259 ± 0.003	0.871 ± 0.001	0.430 ± 0.004	36.22 ± 0.81	36.57 ± 0.73
CT-AGRG [17]	*0.386* ± 0.011	*0.265* ± 0.001	0.863 ± 0.001	*0.488* ± 0.004	*53.80* ± 0.05	*45.27* ± 0.19
**+ clinical indications + demographics**	0.395 ± 0.006	0.268 ± 0.003	0.867 ± 0.001	0.509 ± 0.001	60.43 ± 0.11	48.30 ± 0.05

We used NLG metrics and CE metrics with CRG, recall, and F1 score.

Bold values shows “best results”.

## 7 Conclusion and discussion

In this paper, we present a simple and effective method capable of integrating various sources of information to classify multiple anomalies from chest 3D CT scans, available clinical indications, and age and sex features. We also integrate these information sources for report generation, demonstrating their ability to enhance model performance across various tasks related to 3D CT scans. Furthermore, our experiments validate the effectiveness of each module and the use of a pretrained language model for clinical indication feature extraction. Due to the limited availability of multimodal publicly accessible 3D chest CT datasets, our findings are based solely on the CT-RATE dataset. While this provides a solid foundation for initial validation, reliance on a single dataset may introduce biases related to language patterns, labeling conventions, or demographic representations. Moreover, the demographic features considered in this study (age and sex) remain limited. Future work should therefore aim to include external validation on independent datasets and explore richer metadata to better assess model generalizability and robustness. To enhance multimodal representation of a patient, future work could incorporate additional modalities, such as longitudinal patient data, richer demographic features, or similarity-based retrieval of reports and volumes, to further strengthen multimodal fusion.

## Data Availability

The original contributions presented in the study are included in the article/Supplementary Material; further inquiries can be directed to the corresponding author/s.
